# Orally Active and Selective Tubulin Inhibitors as Anti-Trypanosome Agents

**DOI:** 10.1371/journal.pone.0146289

**Published:** 2016-01-15

**Authors:** Vishal Nanavaty, Rati Lama, Ranjodh Sandhu, Bo Zhong, Daniel Kulman, Viharika Bobba, Anran Zhao, Bibo Li, Bin Su

**Affiliations:** 1 Department of Biology, Geo. & Env. Sciences, College of Sciences and Health Professions, Cleveland State University, 2121 Euclid Avenue, Cleveland, Ohio, 44115, United States of America; 2 Department of Chemistry, College of Sciences and Health Professions, Cleveland State University, 2121 Euclid Avenue, Cleveland, Ohio, 44115, United States of America; 3 Center for Gene Regulation in Health and Disease, College of Sciences & Health Professions, Cleveland State University, 2121 Euclid Avenue, Cleveland, Ohio, 44115, United States of America; University of Hull, UNITED KINGDOM

## Abstract

**Objectives:**

There is an urgent need to develop a safe, effective, orally active, and inexpensive therapy for African trypanosomiasis due to the drawbacks of current drugs. Selective tubulin inhibitors have the potential to be promising drug candidates for the treatment of this disease, which is based on the tubulin protein structural difference between mammalian and trypanosome cells. We propose to identify novel tubulin inhibitors from a compound library developed based on the lead compounds that selectively target trypanosomiasis.

**Methods:**

We used *Trypanosoma brucei brucei* as the parasite model, and human normal kidney cells and mouse microphage cells as the host model. Growth rates of both trypanosomes and mammalian cells were determined as a means to screen compounds that selectively inhibit the proliferation of parasites. Furthermore, we examined the cell cycle profile of the parasite and compared tubulin polymerization dynamics before and after the treatment using identified compounds. Last, *in vivo* anti-parasite activities of these compounds were determined in *T*. *brucei*-infected mice.

**Results:**

Three compounds were selected that are 100 fold more effective against the growth of *T*. *brucei* cells than mammalian cells. These compounds caused cell cycle progression defects in *T*. *brucei* cells. Western analyses indicated that these compounds decreased tubulin polymerization in *T*. *brucei* cells. The *in vivo* investigation revealed that these compounds, when admitted orally, inhibited *T*. *brucei* cell proliferation in mouse blood. However, they were not potent enough to clear up the infection completely.

**Conclusions:**

These compounds are promising lead compounds as orally active agents for drug development of anti-trypanosome agents. A more detail structure activity relationship (SAR) was summarized that will be used to guide future lead optimization to improve the selectivity and potency of the current compounds.

## Introduction

Human African trypanosomiasis, also known as sleeping sickness, is a vector-borne parasitic disease in sub-Saharan Africa with very limited medical resources[1–3]. *Trypanosoma brucei gambiense* (*T*. *b*. *gambiense*) and *Trypanosoma brucei rhodesiense* (*T*. *b*. *rhodesiense*) are the etiological parasites of sleeping sickness in humans. In West and Central Africa, *T*. *b*. *gambiense* is the major parasite that causes the disease, while in sub-Saharan Africa, *T*. *b*. *rhodesiense* predominates. These trypanosome subspecies are responsible for the West and East African forms of the disease, respectively[2]. A third closely related subspecies *Trypanosoma brucei brucei* (*T*. *b*. *brucei*), cannot survive in the human host due to the human serum lytic factor, but is responsible for many cases of nagana in cattle. It significantly impairs the agricultural growth in Africa[4,5]. As *T*. *b*. *brucei* shares many features with *T*. *b*. *gambiense* and *T*. *b*. *rhodesiense* (such as antigenic variation), it is often used as a model for human infections in laboratory and animal studies.

These parasites live and grow extracellularly in the blood and tissue fluids of humans or cattle, and are transmitted among hosts by tsetse flies (*Glossina spp*.). At the beginning of the infection, trypanosomes proliferate in the bloodstream and lymphatic system. Eventually, these parasites cross the blood-brain barrier and enter the central nervous system. At this stage, patients show a variety of neurological symptoms and often exhibit an alteration of the circadian sleep/wake pattern, which is why the disease is called “sleeping sickness”. Without effective treatment, the disease will result in coma and ultimately death. If the patient does not receive treatment before parasites invade the central nervous system, neurological damages caused by parasites are irreversible even after later treatment[3,6]. Therefore, an effective and early treatment is critical.

The current treatment of human trypanosomiasis relies on only four drugs including Suramin, Pentamidine, Melarsoprol and Eflornithine[5]. The main drawbacks of these drugs are: 1) they are toxic to the hosts, which is mainly due to their poor selectivity to parasites than host cells; 2) they can be only administered via intramuscular or intravenous injections; 3) they have very narrow anti-trypanosomiasis spectrum; and 4) their use depends on costly medical resources. Overall, these drugs are not successful in the treatment of the disease, and there is a general lack of effective, inexpensive chemotherapeutic agents for the treatment of human African trypanosomiasis. Therefore, improved chemotherapeutic agents with better selectivity to the trypanosomes are needed to effectively battle this disease[5–7].

Tubulin-containing structures are important for many important cellular functions such cell division, intracellular transport, development and maintenance of cell shape, cell motility, and distribution of molecules on cell surface[8]. Tubulin is a very attractive target in the anti-cancer drug discovery field, and several successful tubulin binders are the first line chemotherapeutic agents in clinic[9]. Tubulin also plays an essential role during trypanosome cell division. The fast population doubling rate of trypanosomes makes them highly dependent on tubulin polymerization/depolymerization[10]. More importantly, tubulin is very critical for trypanosome locomotion, which is an essential function for trypanosomes to survive. Tubulin inhibitors will not only block the *T*. *brucei* cell division but will also affect the locomotion function of flagellum and lead to cell death[11]. The flagellar pocket is known to be an important structure in uptake and internalization of molecules for trypanosomes[12]. Such uptake could enhance the binding of tubulin inhibitors to intracellular tubulin, particularly in the flagella pocket. Therefore, tubulin inhibitors could be effective agents to suppress flagellar locomotion function[11]. These factors indicate that there are advantages of tubulin inhibitors for the treatment of trypanosomiasis.

Tubulin is a highly conserved protein. However, differences in susceptibility to antimitotic agents are known to exist between tubulins from different organisms, indicating that differences of tubulin structures exist among different species[13]. Based on the differences of tubulin in *T*. *brucei* and mammalian cells, it is highly expected that selective tubulin inhibitors could be developed. We have developed a class of tubulin inhibitors as anti-cancer agents[14,15]. These compounds share the same core scaffold, and bind to colchicine-binding domain on tubulin[14]. Based on differences of the binding pocket between mammalian and *T*. *brucei* tubulins, we examined the growth of *T*. *brucei* and mammalian cells treated with our compounds as a screening test[16]. Some compounds exhibited very specific inhibitory effect on *T*. *brucei* growth, with selectivity index (IC_50_ inhibiting human cell growth/IC_50_ inhibiting *T*. *brucei* cell growth) being 5 or more. More importantly, the pharmacophore of these compounds enhancing the mammalian cell growth inhibition was different to the pharmacophore promoting the *T*. *brucei* cell growth inhibition. In the current study, we screened a compound library including 79 derivatives developed based on previous lead compounds. More selective tubulin inhibitors were identified and they exhibited potent tubulin polymerization inhibition in *T*. *brucei* cells and also caused defective cytokinesis. Furthermore, selected candidates showed *in vivo* activities to inhibit *T*. *brucei* replication in infected mice when administered orally.

## Materials and Methods

### Reagents and animals

**Fig 1:**
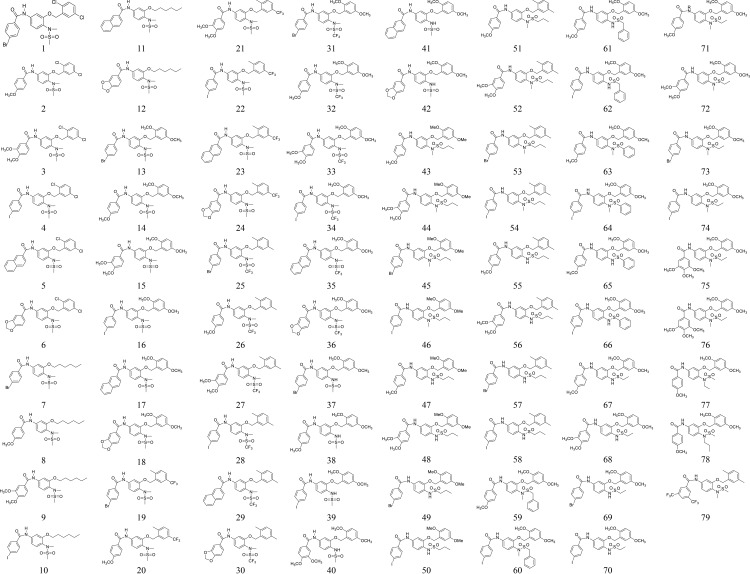
Structures of the compounds.

Sulfonamide tubulin inhibitors were synthesized in our laboratory and the procedures were described in previous studies[17,18]. All the structures of the compounds are listed in Fig 1.

3-(4,5-dimethylthiazol-2-yl)-5-(3-carboxymethoxyphenyl)-2-(4-sulfophenyl)-2H- tetrazolium (MTS) reagents were from Promega life science (Madison, WI). 3-(4, 5-dimethylthiazol-2-yl)-2, 5-diphenyl-2H-tetrazolium bromide (MTT) and sesame oil were from Sigma-Aldrich (Milwaukie, WI). 3 month old female CD1 mice were purchased from Charles River laboratory.

## Cell culture

HEK293 kidney cells, mouse macrophage RAW267.4 cells were obtained from ATCC (Rockville, MD) and maintained in RPMI1640 medium supplemented with 10% fetal bovine serum (FBS), 2 mmol/L L-Glutamine, 1 mmol/L sodium pyruvate, and 100 U/mL penicillin-streptomycin. FBS was heat inactivated for 30 min at 56°C. Mammalian cells were grown at 37°C, in a Heraeus water-jacketed incubator with 5% CO_2_. *T*. *b*. *brucei* Lister 427 cells were cultured in HMI-9 medium [19] with 10% FBS at 37°C in a Heraeus water-jacketed incubator with 7.5% CO_2_.

### Mammalian cell viability analysis

The MTT assay was used to examine the effect of tubulin inhibitors on HEK293 and RAW267.4 cell growth with quadruplication. Cells were grown in RPMI1640 medium in 96-well flat-bottomed plates for 24 hrs and were exposed to various concentrations of test compounds dissolved in DMSO (final concentration ≤0.1%) in medium for 48 hrs. Controls received DMSO at a concentration the same as that in drug-treated cells. Cells were incubated in 200 μl of 0.5 mg/ml of MTT reagent diluted in fresh media at 37°C for 2 hrs. Supernatants were removed from the wells, and the reduced MTT dye was solubilized in 200 μl/well DMSO. Absorbance at 570 nm was determined on a SpectraMax Plus384 spectrophotometer (Molecular Devices). Data obtained with quadruplication were normalized and fitted to a dose–response curve using GraphPad Prism v.5 (GraphPad), and standard deviations were used.

### *T*. *brucei* cell viability analysis

The MTS assay was used to examine the effect of tubulin inhibitors on *T*. *b*. *brucei* cell viability[16]. 5000 cells of *T*. *brucei* were seeded in 96 well plates and treated with 0.1% DMSO and tested agents at various concentrations for 48 hrs at 37°C. Subsequently, 20 μL of MTS (5% PMS) from the CellTiter Cell Proliferation Assay (Promega) was added to 200 μL of *T*. *b*. *brucei* culture in each well and incubated at 37°C for an 3 hrs. Soluble formazan, produced by viable cells due to reduction of MTS, was measured at 490 nm with a SpectraMax Plus384 spectrophotometer (Molecular Devices). Data obtained with quadruplication were normalized and fitted to a dose–response curve using GraphPad Prism v.5 (GraphPad), and standard deviations were used.

### Fluorescence Activated Cell Sorting (FACS) analysis

*T*. *brucei* cells were incubated with 0.5% DMSO (as a control), compound **79** (500 nM), compound **11** (500 nM), and **12** (500 nM) for 7 hrs. Subsequently, 0.1 million cells were harvested at 1500 rpm for 10 mins at 4°C, washed twice with 1 x PBS/2 mM EDTA, and resuspended in 200 μl of 1 x PBS/2 mM EDTA per 0.1 million cells in 15 ml conical tubes. Cells were then fixed by adding 2 mL of icy-cold 70% EtOH during vortexing followed by incubating at 4°C for a minimum of 16 hrs. Cell pellets were collected by centrifugation at 1,000 rpm for 5 mins at 4°C and stained with 0.5 mL of staining solution (950 μl of 1xPBS/2mM EDTA, 20 μl of RNAse A (10 mg/ml), 50 μl of propidium iodide (1 mg/ml)) at 37°C for 30 mins and filtered for the Fluorescence Activated Cell Sorting (FACS) analysis. FACS was done on FACS Aria II (BD Biosciences) and data from three independent experiments were analyzed using the Flowjo software.

### *T*. *brucei* cell lysate preparation and Western blotting

*T*. *brucei* cells were incubated with 0.5% DMSO, compound **79** (500 nM), compound **11** (500 nM), and **12** (500 nM) for 24 hrs. Cell pellets were harvested by centrifugation 1,500 rpm for 10 mins at 4°C, washed twice with 1X TDB buffer (5 mM KCl, 80 mM NaCl, 1 mM MgSO_4_, 20 mM Na_2_HPO_4_, 2 mM NaH_2_PO_4_, 20 mM glucose, pH 7.4) with one complete protease inhibitor tablet (Roche), and lysed with 300 μl of lysis buffer (80 mM Pipes, pH 6.8, 1 mM EGTA, 1 mM MgCl_2_, 0.2% Triton X-100, 10% glycerol and one complete protease inhibitor tablet (Roche)) at 30°C for 5 mins. The cell lysate was centrifuged at 2,000 rpm for 10 mins at 4°C, and the supernatant was transferred into a fresh Eppendorf tube. Pellets were re-suspended in lysis buffer and sonicated. 50 μl of 2 x SDS buffer was added to equal volume of cell lysate and re-suspended pellet fraction, which were boiled at 95°C for 5 mins. Protein lysates from equal number of cells were separated on 10% polyacrylamide gels by electrophoresis. Proteins were transferred onto nylon hyblot CL membranes. Tubulin antibody (TAT-1, a gift from Dr. K. Gull) was used in the following western analysis. The experiments were repeated three times and the representative bands are listed.

### *In vivo* testing

The animal study was approved by Cleveland State University animal care and use committee and adheres to the Guide for the Care and Use of Laboratory Animals, 8^th^ edition (NIH). 3 month old female mice (Charles River) were randomly divided to three groups (4 mice per group) and infected with 100,000 *T*. *brucei* cells per mouse via intraperitoneal injection. On the second day, mice were started to be treated daily via oral gavage with individual compounds (compound **79** at a dose of 400 mg/kg and compound **12** at a dose of 200 mg/kg) using the sesame oil formulation (200uL per mouse as administration) for four days. The negative control group received sesame oil and vehicle DMSO. We also used Pentamidine (20mg/kg ip injection once) as a positive control and it totally cleared up the infection by the end of the treatment with only one injection on the second day after infection. Due to the different administration routes, the results of Pentamidine were not listed. Start on day 3, 5 μl of tail blood was collected daily for *T*. *brucei* cell counting. More specifically, the *T*. *brucei* cells in the blood was diluted 200 times with TDB buffer and counted with a hemocytometer. The parasitemia level was evaluated by the *T*. *brucei* cell number in the blood based on the counting. Mice were euthanized with isoflurane overdose when parasitemia reached more than 200 million cells/mL in blood. Mice weight was examined before and after treatment.

## Results and Discussion

### Compound screening identified three candidates as the most potent and selective ones to inhibit *T*. *brucei* growth

We examined inhibitory activities of tubulin inhibitors synthesized in our laboratory using a cell proliferation assay. *T*. *b*. *brucei* Lister 427 cells were used as the parasite model, and human normal kidney cells HEK293 and mouse macrophage RAW267.4 cells were used as the mammalian cell control.

Results of cell growth inhibition are shown in Table 1.

**Table 1:** Comparison of the growth inhibitory effects of the tubulin inhibitors on mammalian and *T*. *brucei* cells (IC_50_ ± standard deviations are used to indicate the potency of the compounds).

Comp	IC_50_ against *T.brucei* cell growth (μM)	IC_50_ against macrophage RAW267.4 cell growth (μM)IC_50_	IC_50_ of macrophage cells /IC_50_ of *T. brucei*	IC_50_ against HEK293 cell growth (μM)	IC_50_ of HEK293 cells /IC_50_ of *T. brucei*
1	0.77 ± 0.29	2.80 ± 1.81	3.6	1.53 ± 0.65	2
2	4.08 ± 1.96	1.09 ± 0.75	0.3	0.39 ± 0.15	0.1
3	4.41 ± 2.41	2.60 ± 1.74	0.59	1.74 ± 0.77	0.4
4	0.31 ± 0.15	0.52 ± 0.34	1.7	0.31 ± 0.11	1
5	1.64 ± 0.55	1.26 ± 0.63	0.8	21.60 ± 12.10	13.2
6	7.98 ± 3.39	3.31 ± 2.09	0.4	15.80 ± 7.79	2
7	0.99 ± 0.28	121.20 ±58.08	122.4	74.90 ± 33.20	75.7
8	1.09 ± 0.43	10.10 ± 4.65	9.3	41.40 ± 16.40	38
9	3.39 ± 1.22	65.90 ± 25.30	19.4	50.90 ± 23.10	15
10	1.68 ± 0.84	11.20 ± 7.01	6.7	8.53 ± 2.86	5.1
11	1.16 ± 0.62	>200.0	>172.4	195.0 ± 146.0	168.1
12	0.82 ± 0.41	>200.0	>243.9	192.0 ± 129.0	234.1
13	1.25 ± 0.44	0.012 ± 0.008	0.01	0.025± 0.012	0.02
14	7.17 ± 3.42	0.001± 0.0005	0.0001	0.001±0.0004	0.0001
15	3.19 ± 1.42	0.067 ± 0.051	0.02	0.031 ± 0.019	0.01
16	0.79 ± 0.32	0.007± 0.0004	0.01	0.004 ± 0.001	0.05
17	2.49 ± 1.54	0.009± 0.005	0.004	0.005 ± 0.002	0.002
18	10.0 ± 4.89	0.004 ± 0.002	0.0004	0.008 ± 0.003	0.0008
19	2.37 ± 0.96	3.15 ± 1.97	1.3	11.20 ± 4.87	4.7
20	3.46 ± 1.85	1.21 ± 0.58	0.3	2.73 ± 1.34	0.8
21	6.35 ± 2.30	1.90 ± 0.90	0.3	3.48 ± 1.56	0.5
22	0.80 ± 0.24	1.94 ± 1.12	2.4	0.80 ± 0.37	1
23	2.15 ± 0.61	4.98 ± 2.61	2.3	0.86 ± 0.26	0.4
24	2.40 ± 0.96	4.67 ± 2.33	1.9	8.99 ± 3.54	3.7
25	0.47 ± 0.17	8.98 ± 4.65	19.1	6.36 ± 3.99	13.5
26	1.29 ± 0.51	3.79 ± 1.83	2.9	5.85 ± 3.97	4.5
27	3.91 ± 2.27	12.70 ± 6.89	3.2	1.63 ± 0.80	0.4
28	0.73 ± 0.38	2.57 ± 1.53	3.5	2.81 ± 1.02	3.8
29	1.07 ± 0.44	30.76 ± 13.67	28.7	27.90 ± 6.45	26.1
30	2.25 ± 0.77	9.91 ± 4.82	4.4	5.88 ± 2.53	2.6
31	0.37 ± 0.15	0.52 ± 0.32	1.4	0.41 ± 0.09	1.1
32	0.89 ± 0.41	0.17 ± 0.08	0.2	0.15 ± 0.05	0.2
33	2.09 ± 0.98	0.48 ± 0.27	0.2	0.79 ± 0.26	0.4
34	0.34 ± 0.16	0.18 ± 0.09	0.5	0.14 ± 0.06	0.4
35	0.50 ± 0.11	1.25 ± 0.83	2.5	0.76 ± 0.26	1.5
36	1.12 ± 0.39	0.42 ± 0.23	0.4	0.26 ± 0.09	0.5
37	8.54 ± 4.26	0.051 ± 0.030	0.006	0.061± 0.026	0.007
38	72.90± 2.20	0.010 ± 0.005	0.0001	0.011 ± 0.007	0.0002
39	9.13 ± 4.39	0.039 ± 0.019	0.004	0.028 ± 0.013	0.003
40	14.0 ± 4.88	0.018 ± 0.010	0.001	0.009 ± 0.004	0.0006
41	190.90±138.50	0.021±0.011	0.0001	0.013 ± 0.006	0.00007
42	96.20 ± 56.90	0.016±0.008	0.0002	0.015 ± 0.006	0.0002
43	2.69 ± 0.89	0.016±0.011	0.006	0.014 ± 0.005	0.005
44	2.04 ± 1.02	0.043±0.017	0.02	0.005 ± 0.002	0.002
45	0.92 ± 0.23	0.055±0.027	0.06	0.006 ± 0.003	0.007
46	0.32 ± 0.10	0.007±0.003	0.02	0.005 ± 0.002	0.02
47	22.60 ± 9.85	0.11 ± 0.05	0.005	0.12 ± 0.05	0.005
48	10.60 ± 4.89	0.23 ± 0.07	0.02	0.20 ± 0.06	0.02
49	1.48 ± 0.59	0.072±0.033	0.05	0.41 ± 0.19	0.3
50	1.31 ± 0.52	0.065±0.037	0.05	0.070 ± 0.020	0.05
51	2.29 ± 1.11	0.20 ± 0.090	0.09	1.31 ± 0.35	0.6
52	2.53 ± 1.37	1.99 ± 1.03	0.8	1.23 ± 0.42	0.5
53	0.56 ± 0.21	0.83 ± 0.51	1.5	0.69 ± 0.29	1.8
54	0.36 ± 0.13	0.18 ± 0.09	0.5	0.06 ± 0.02	0.2
55	9.43 ± 4.42	2.25 ± 0.87	0.2	1.96 ± 0.99	0.2
56	11.90 ± 5.84	10.35 ± 3.82	0.9	6.61 ± 2.02	0.6
57	2.13 ± 0.96	13.10 ± 5.51	6.2	15.90 ± 8.43	7.5
58	1.73 ± 0.68	1.74 ± 0.61	1	1.26 ± 0.73	0.7
59	1.04 ± 0.48	0.015±0.009	0.01	0.019 ± 0.009	0.01
60	0.20 ± 0.08	0.010±0.004	0.05	0.010 ± 0.005	0.05
61	1.85 ± 1.12	0.39 ± 0.21	0.2	0.17 ± 0.07	0.1
62	1.41 ± 0.75	0.29 ± 0.16	0.2	0.18 ± 0.08	0.1
63	2.33 ± 0.96	0.064±0.030	0.03	0.18 ± 0.09	0.3
64	0.54 ± 0.16	0.06± 0.03	0.1	0.06 ± 0.02	0.1
65	4.42 ± 1.31	1.25 ± 0.50	0.3	2.25 ± 1.02	0.5
66	2.12 ± 0.76	0.56 ± 0.21	0.3	0.75 ± 0.40	0.4
67	43.50 ± 23.50	0.044±0.019	0.001	0.027 ± 0.012	0.0006
68	45.90 ± 26.50	0.076±0.037	0.002	0.065 ± 0.028	0.001
69	1.34 ± 0.68	0.085±0.047	0.06	0.049 ± 0.022	0.04
70	1.19 ± 0.53	0.037±0.017	0.03	0.036 ± 0.011	0.03
71	31.50 ± 15.60	0.007±0.003	0.0002	0.004 ± 0.002	0.0001
72	3.02 ± 2.30	0.057±0.030	0.02	0.049 ± 0.021	0.02
73	1.88 ± 1.12	0.044±0.023	0.02	0.012 ± 0.004	0.006
74	0.41 ± 0.19	0.006±0.002	0.01	0.004 ± 0.002	0.01
75	3.45 ± 1.25	0.21 ± 0.12	0.06	0.11 ± 0.04	0.03
76	1.95 ± 1.16	0.23 ± 0.12	0.1	0.11 ± 0.05	0.06
77	2.43 ± 1.47	0.010±0.004	0.004	0.020± 0.012	0.008
78	2.01 ± 1.13	0.95 ± 0.66	0.5	0.73 ± 0.36	0.4
79	0.68 ± 0.13	69.55±23.36	102.3	92.36 ± 43.25	135.8

The selective index is calculated by dividing the IC_50_s of the mammalian cell growth inhibition by the IC_50_s of the *T*. *brucei* cell growth inhibition.

**Fig 2:**
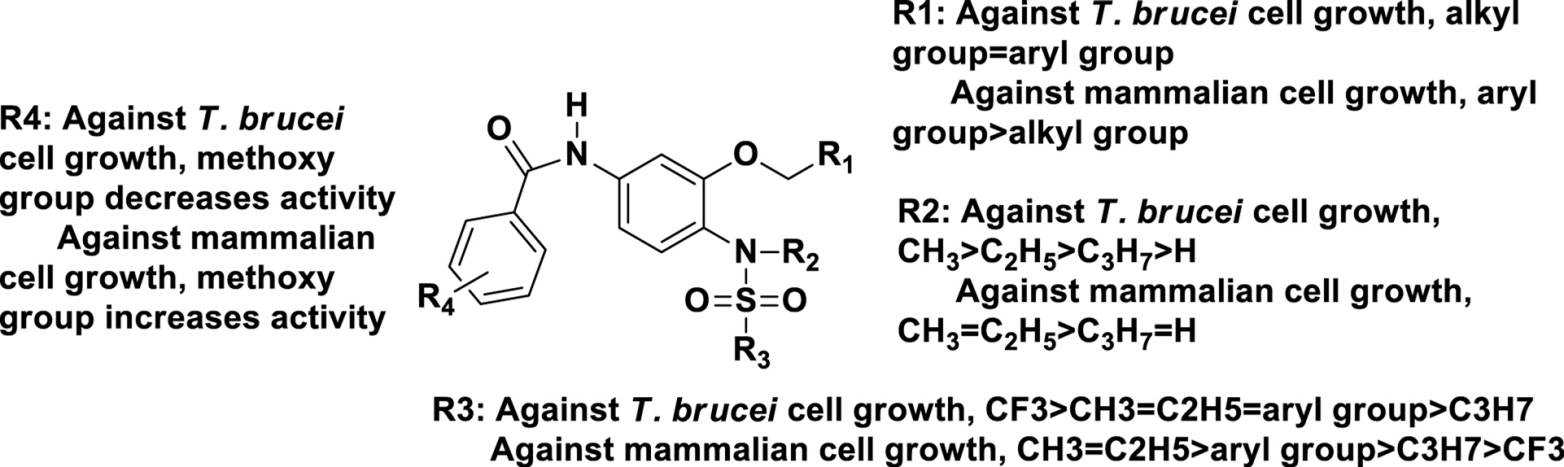
SAR results of the anti-mammalian cell growth activity and anti-*T*. *brucei* growth activity of the sulfonamide tubulin inhibitors.

For the four moieties (R1 –R4) of the compound scaffold, various functional groups were introduced to enhance the anti-trypanosome activity and decrease anti-mammalian cell activity (Fig 2).

For R1 domain, some analogs were designed with alkyl and aryl groups as the substituents. To increase the structural variability, we also set electron-withdrawing and donating group substituted aryl group as R1 moiety. Based on IC_50_s of the mammalian cell growth inhibition, aryl groups are preferred to target mammalian cells. Particularly, the 2, 5-dimethylbenzyl moiety and 2, 5-dimethoxybenzyl group dramatically increased the inhibition of mammalian cell proliferation. Several derivatives (compounds **13**–**18**, **38**–**43**) inhibited the proliferation of HEK293 and RAW267.4 cells with IC_50_ values at subnanomolar level (Table 1), which is also consistent to our previous anti-cancer investigation of these compounds[17,18]. Obviously, methoxy group is not a good substituent on this scaffold for anti-trypanosome agents, because it is expected to increase the toxicity to host cells. The Alkyl group such as hexyl at the R1 moiety (compounds **7–12**) significantly decreased the inhibition to mammalian cell growth, and it has the similar activity against *T*. *brucei* cell proliferation as the aryl substituted analogs. Electron-withdrawing group substituted aryl moiety (compounds **19–24**) decreased the anti-mammalian cell activity compared to the electron-donating group (compounds **13–18**), but did not affect the anti-trypanosome activity significantly. Therefore, it makes sense to synthesize more alkyl substituent groups and electron-withdrawing group bonded aryl groups at R1 moiety in future optimization.

For R2 moiety, it seems that a middle sized alkyl group benefits both inhibitions to mammalian and *T*. *brucei* cell growth. Compounds **37–42**, **67–70** exhibited lower potency than their N-methyl derivatives **13–18** and **71–74**, respectively. This result indicates that hydrogen is the least preferred substituent at R2. On the other hand, methyl group promoted the activity against all mammalian and *T*. *brucei* cells. More bulky groups are not preferred either, because ethyl and propyl groups decreased the activity in all tests compared to corresponding methyl analogs. Therefore, it is difficult to increase the selectivity of the compounds to *T*. *brucei* cells by modifying R2 moiety.

Fortunately, R3 moiety is a good domain to increase the selectivity of the compounds. Replacing methyl sulfonamide group in these compounds with trifluoromethyl sulfonamide group (compounds **25–36**) significantly impaired their activity against mammalian cell proliferation, suggesting that the trifluoromethyl sulfonamide group was well tolerated by the mammalian cells. Unexpectedly, this modification increased the anti-trypanosome activity simultaneously, indicated by IC_50_s of compounds **13–18** verse **31–36.** All trifluoromethyl analogs showed better activity against *T*. *brucei* cell growth than their corresponding methyl analogs. In terms of bulkiness factor, methyl and ethyl groups showed similar activities in both parasitic and mammalian cells, and propyl group decreased both activities. Arylsulfonamides and alkyl/arylsulfonamides (**59–66**) all showed potent activity against *T*. *brucei* cells with low micromole IC_50_s. However, the greatly reduced water solubility of these compounds limits further optimization to any other aryl substituent at R3 domain. Overall, to increase the activity and selectivity of the compound for the inhibition of *T*. *brucei* cell growth, trifluoromethyl is the best fit for R3, and it will be included in the future optimization.

At R4 moiety, methoxy groups impaired the anti-trypanosome activity but increased the activity against mammalian cells, which is represented by compounds **14** and **38**. Halogen atom iodine improved the activity to against both *T*. *brucei* and mammalian cells, which is indicated by compounds **16, 22, 28**, and **46**. We haven’t identified any group at R4 domain that would only increase the anti-trypanosome activity in the current investigation. However, our previous study suggested that electron-withdrawing group such as trifluoromethyl might increase the selectivity to inhibit *T*. *brucei* cell growth[16]. In the future, further optimization will be mainly focused on R1 and R4 moieties, R2 will be the methyl group and R3 will be the trifluoromethyl group. Based on the selective index (we used 100 as the cutoff for selection), we selected compounds **11**, **12** and **79** for the following anti-trypanosomal mechanism investigation and *in vivo* activity determination.

### Three compounds inhibit tubulin polymerization and affect *T*. *brucei* cell cycle progression

**Fig 3:**
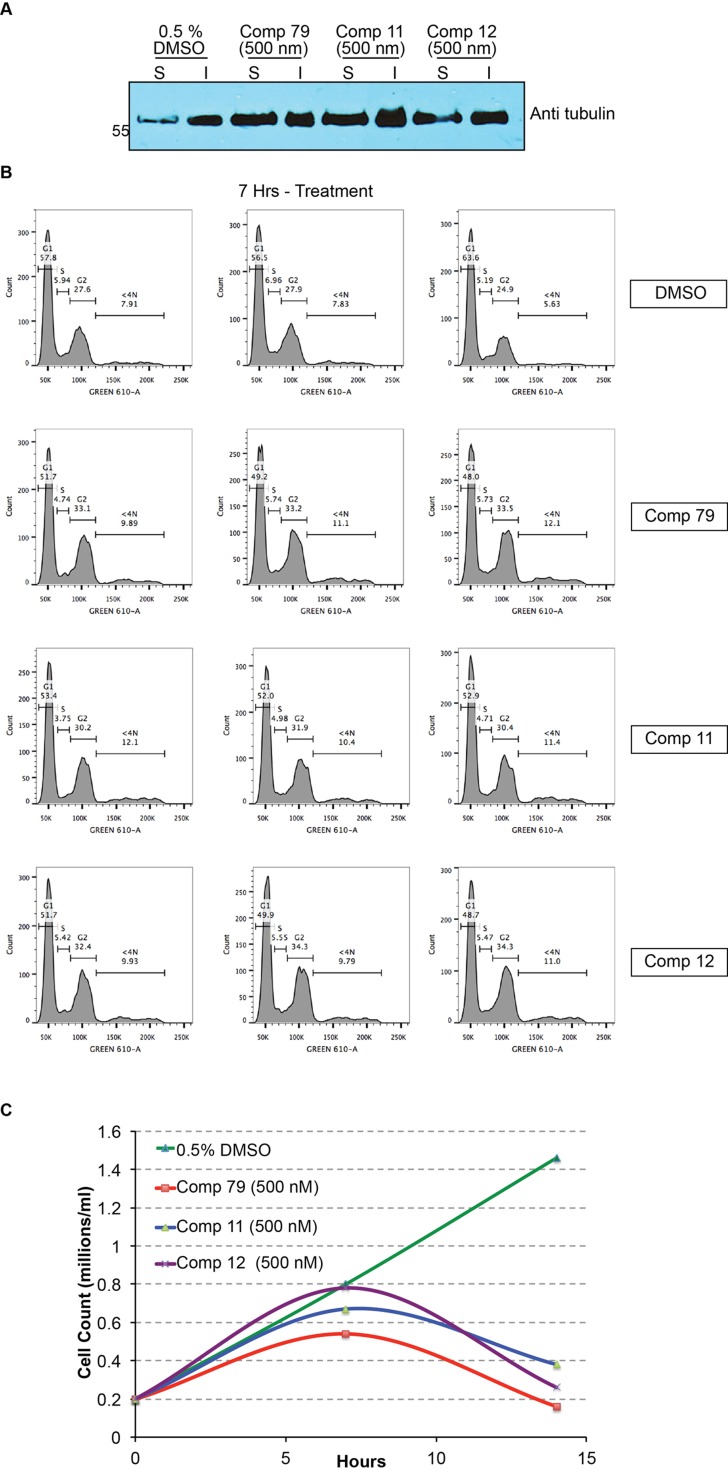
Tubulin inhibitors cause defective cell cycle progression and inhibit tubulin polymerization. FACS, Western blot and cell growth are all from three independent experiments. (A) *T*. *brucei* cells were treated with 0.5% DMSO, compound **79** (500 nM), compound **11** (500 nM), and **12** (500 nM) for 24 hrs, the soluble tubulin and non-soluble microtubules are examined with western blot. The level of soluble tubulin (non-polymerized tubulin) is significantly increased. (B) *T*. *brucei* cells were treated with 0.5% DMSO, compound **79** (500 nM), compound **11** (500 nM), and **12** (500 nM) for 7 hrs. The cells were harvested and stained with propidium iodide, then examined with FACS analysis. Compounds treatment causes defective cell cycle progression. (C) Compounds treatment significantly decreases cell division. *T*. *brucei* cells were treated with 0.5% DMSO, compound **79** (500 nM), compound **11** (500 nM), and **12** (500 nM) for 14 hrs. At 7hrs and 14hrs, cells were collected and the cell number was counted with a hemocytometer.

Our current compounds were developed based on our previous results, and the lead compounds were speculated to be selective tubulin inhibitors for *T*. *brucei* cells[16]. Therefore, we examined if the selected new drug candidates could affect cell cycle progression in *T*. *brucei* cells. Three compounds (**11, 12**, and **79**) at 0.5 μM significantly decreased cell proliferation after 7 hrs treatment and led to cell death at later stage (Fig 3).

FACS analysis of cells treated for 7 hrs showed that there were significantly more G2/M phase cells in the population, although this increase was mild. This suggests that cells may spend a longer time during G2/M, which is consistent with the idea that mitosis progression is delayed. We also observed significantly more cells carrying 4C and 8C DNA contents among the cell population (Fig 3 and Table 2).

**Table 2:** *T*. *brucei* cell distribution in different phases after the compound treatment. Compared to the control, all three compounds cause cell cycle progression defects in *T*. *brucei* cells. FACS results from three independent experiments (mean ± standard deviation) are given.

					(Treatment vs DMSO)	
	G1	S	G2/M	>4N	P value of G2/M	P value of >4N
DMSO	59.20±3.61	6.03±0.89	26.80±1.65	7.12±1.29		
Comp 79(0.5μM)	49.63±1.89	5.40±0.57	33.27±0.21	11.03±1.11	0.020	0.017
Comp 11(0.5μM)	52.77±0.71	4.48±0.65	30.83±0.93	11.30±0.85	0.032	0.013
Comp 12(0.5μM)	50.10±1.51	5.48±0.07	33.67±1.10	10.24±0.66	0.006	0.034

It is known that bloodstream form *T*. *brucei* cells have a cytokinesis checkpoint where cell division is blocked but DNA replication can continue, leading to accumulation of 4C and 8C cells[20]. Our FACS analysis gave the same cell cycle profile as cells in cytokinesis checkpoint, indicating that treating *T*. *brucei* cells with the tubulin blocker candidates caused cytokinesis defects[20]. After 14 hrs treatment, cell death was significantly increased (Fig 3C), which prevented us from performing the FACS analysis. The significant cell death also indicates that the compounds might interfere with other cell machinery to impact cell viability as well.

Our results suggest that the compounds mainly interfered with the microtubule dynamics. To test this hypothesis, we determined the amount of the polymerized and the un-polymerized tubulin after treating *T*. *brucei* cells with the same compounds. We found that all three compounds increased the amount of soluble tubulin (Fig 3A), indicating that after the treatment, the soluble tubulin dimers were not efficiently converted to the insoluble tubulin polymers. Therefore, our compounds likely inhibited tubulin polymerization in *T*. *brucei* cells, which in turn caused a delay of G2/M progression and defective cytokinesis. They are a group of novel *T*. *brucei* selective tubulin inhibitors.

### Compounds 12 and 79 are orally active in *in vivo* studies using *T. brucei-*infected mice

**Fig 4:**
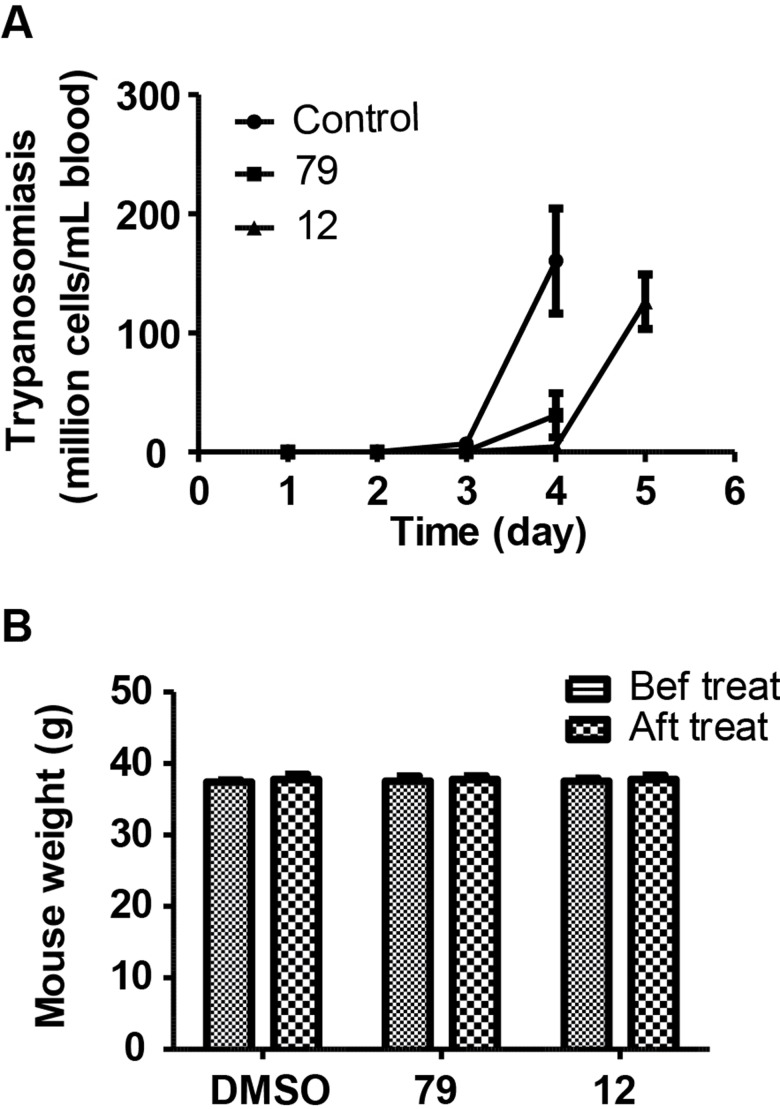
*In vivo* testing of selective tubulin inhibitors. Mice were randomly divided to three groups (4 mice per group) and infected with 100,000 *T*. *brucei* cells per mouse via intraperitoneal injection. Subsequently, mice were treated daily via oral gavage with individual compounds (compound **79** at a dose of 400 mg/kg and compound **12** at a dose of 200 mg/kg) using the sesame oil formulation. The control group received sesame oil and vehicle DMSO. (A) *T*. *brucei* cell count of mouse tail blood after mice were treated with our compounds. (B) Mouse weight change before and after the treatment.

For drug discovery, the most critical part is the drug’s *in vivo* activity. We used an acute infection mouse model to investigate the *in vivo* activities of our identified compounds. The current clinical drugs used to treat *T*. *brucei* infection are not orally active, and our focus is to develop orally active drug candidates for the treatment. Therefore, the three drug candidates were administered to the infected mice via oral gavage. When we prepared the drug formulation, the low solubility of compound **11** limited its administration and was eliminated from the following assays. Compounds **12** and **79** both exhibited inhibitory activity to *T*. *brucei* proliferation in mouse blood (Fig 4).

However, they cannot clear *T*. *brucei* infection completely, as *T*. *brucei* cell counts eventually went up. There are several possible explanations for this observation. First, our compounds may have low bio-availability, and very low percentage of drug can reach the portal vain after oral gavage. Second, these compounds may have high first pass metabolism, and the original drug lose its activity after being released from the liver. Third, these compounds may have high serum protein binding affinity, and they cannot be effectively up taken by *T*. *brucei* cells. Nevertheless, the two selective tubulin inhibitors showed oral activities to inhibit *T*. *brucei* growth in the animal model. Further optimization to increase the *in vivo* activities of these compounds is needed in the future.

## Conclusion

African trypanosomiasis is a threat to public health in sub-African regions. Without effective vaccines and satisfactory drug treatments, the development of new drugs for the disease is urgently needed. Our research leads to a unique molecular scaffold that selectively targets *T*. *brucei* tubulin, and opens a new area on trypanosome-specific tubulin inhibitor development. Based on the inhibitory effects of our compounds on *T*. *brucei* cell proliferation and mammalian cell growth, a SAR is summarized. The pharmacophore of the tubulin inhibitor promoting the mammalian cell growth inhibition and the functional groups enhancing the parasite growth inhibition are described in Fig 2. Furthermore, three highly selective tubulin inhibitors were identified, and they interfered with tubulin polymerization in *T*. *brucei* cells, resulting in defective cell cycle progression and increased soluble tubulin dimer levels in cell cytosol. In addition, two drug candidates showed oral activities to block *T*. *brucei* cell proliferation in the mouse model, indicating that these compounds are good leads for further optimization. It is therefore expected that more selective and potent tubulin inhibitors for trypanosome infections could be developed based on this discovery.
